# Spatial Distribution and Burden of Emerging Arboviruses in French Guiana

**DOI:** 10.3390/v13071299

**Published:** 2021-07-02

**Authors:** Sarah Bailly, Dominique Rousset, Camille Fritzell, Nathanaël Hozé, Sarrah Ben Achour, Léna Berthelot, Antoine Enfissi, Jessica Vanhomwegen, Henrik Salje, Sandrine Fernandes-Pellerin, Mona Saout, Anne Lavergne, Jean-Claude Manuguerra, Jean-François Carod, Félix Djossou, Simon Cauchemez, Claude Flamand

**Affiliations:** 1 Epidemiology Unit, Institut Pasteur in French Guiana, 97300 Cayenne, French Guiana; sbailly@pasteur-cayenne.fr (S.B.); camillefritzell@gmail.com (C.F.); mona.saout@univ-guyane.fr (M.S.); 2 Arbovirus National Reference Center, Institut Pasteur in French Guiana, 97300 Cayenne, French Guiana; drousset@pasteur-cayenne.fr (D.R.); sarrahbenachour@gmail.com (S.B.A.); berthelot.lena@gmail.com (L.B.); aenfissi@pasteur-cayenne.fr (A.E.); alavergne@pasteur-cayenne.fr (A.L.); 3 Mathematical Modelling of Infectious Diseases Unit, Institut Pasteur, UMR2000, CNRS, 75015 Paris, France; nathanael.hoze@pasteur.fr (N.H.); hsalje@gmail.com (H.S.); simon.cauchemez@pasteur.fr (S.C.); 4 Environment and Infectious Risks Unit, Institut Pasteur, 75015 Paris, France; jessica.vanhomwegen@pasteur.fr (J.V.); jean-claude.manuguerra@pasteur.fr (J.-C.M.); 5 Department of Genetics, University of Cambridge, Cambridge CB2 3EH, UK; 6 Clinical Coordination of Translational Research Center, Institut Pasteur, 75015 Paris, France; sandrine.fernandespellerin@pasteur.fr; 7 Medical Laboratory, Centre Hospitalier de l’Ouest Guyanais, 97320 Saint-Laurent du Maroni, French Guiana; jf.carod@ch-ouestguyane.fr; 8 Infectious and Tropical Diseases Unit, Centre Hospitalier Andrée Rosemon, 97300 Cayenne, French Guiana; felix.djossou@ch-cayenne.fr

**Keywords:** arboviruses, Dengue, Zika, Chikungunya, Mayaro, prevalence studies, French Guiana

## Abstract

Despite the health, social and economic impact of arboviruses in French Guiana, very little is known about the extent to which infection burden is shared between individuals. We conducted a large multiplexed serological survey among 2697 individuals from June to October 2017. All serum samples were tested for IgG antibodies against DENV, CHIKV, ZIKV and MAYV using a recombinant antigen-based microsphere immunoassay with a subset further evaluated through anti-ZIKV microneutralization tests. The overall DENV seroprevalence was estimated at 73.1% (70.6–75.4) in the whole territory with estimations by serotype at 68.9% for DENV-1, 38.8% for DENV-2, 42.3% for DENV-3, and 56.1% for DENV-4. The overall seroprevalence of CHIKV, ZIKV and MAYV antibodies was 20.3% (17.7–23.1), 23.3% (20.9–25.9) and 3.3% (2.7–4.1), respectively. We provide a consistent overview of the burden of emerging arboviruses in French Guiana, with useful findings for risk mapping, future prevention and control programs. The majority of the population remains susceptible to CHIKV and ZIKV, which could potentially facilitate the risk of further re-emergences. Our results underscore the need to strengthen MAYV surveillance in order to rapidly detect any substantial changes in MAYV circulation patterns.

## 1. Introduction

Arboviral diseases are caused by viruses that are transmitted to humans through the bite of an infected arthropod, primarily mosquitoes and ticks. Although arboviruses have been infecting humans for a long time, in recent decades they have become a growing public health problem due to the emergence and re-emergence of the diseases they cause throughout the world [1,2,3,4,5,6,7,8]. They are initially transmitted in zoonotic cycles involving nonhuman primates and arboreal mosquitoes, but can enter human-to-human cycles involving urban transmission that now extends beyond tropical and subtropical regions. This resurgence of arboviruses may be explained in part by the increase in international travel and globalization, which has continued to accelerate the introduction of arboviruses into new areas and their geographic expansion [1,9,10].

**Figure 1:**
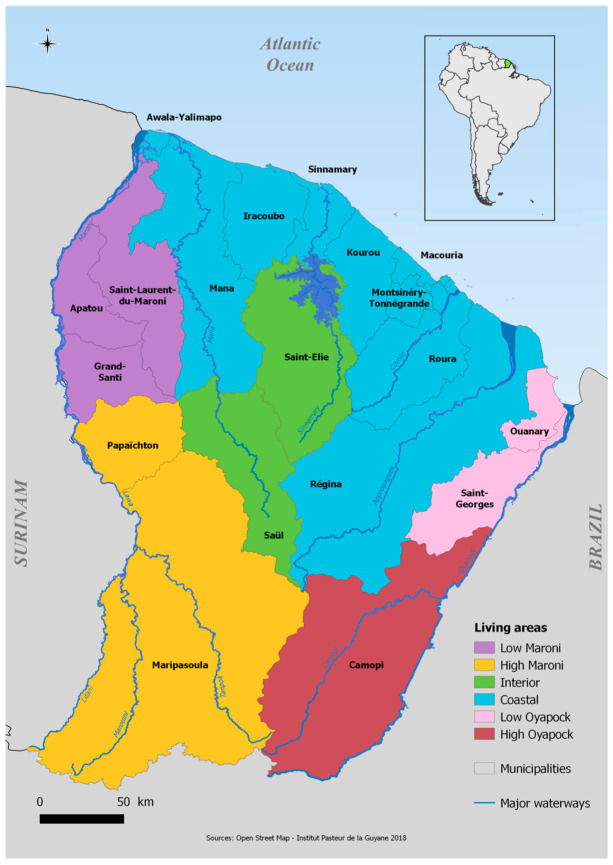
Map of French Guiana with geographical areas.

In Latin American and Caribbean countries, the reintroduction and dissemination of *Ae. aegypti* took place in the 1970s [11], leading to a progressive increase in the risk of arboviruses and the regular occurrence of large-scale epidemics [11,12,13]. French Guiana, a French overseas territory of 290,000 inhabitants, has been confronted for many decades with the transmission of several arboviruses, some of which have caused epidemics affecting almost the entire territory [14,15,16,17,18,19,20,21,22,23]. The territory is composed of an urbanized coastal strip along the Atlantic Ocean, totally accessible by road, where 90% of the population lives, and more remote areas located in the middle of the Amazonian forest zone (called the interior zone) or along the Surinamese (Low and High Maroni) and Brazilian (Low and High Oyapock) borders (Figure 1).

Since the first cases of dengue hemorrhagic fever (DHF) reported in 1992 [22], *Ae. aegypti* mosquitoes have been responsible for several major dengue fever outbreaks [14,15] and for the chikungunya outbreak between February 2014 and October 2015 [19,24]. After rapidly rising from obscurity to a public health emergency of international concern following its appearance in Brazil, Zika virus (ZIKV) caused a major epidemic between January and September 2016 [17]. Cases of Mayaro virus, which is considered as an emerging virus transmitted by *Haemagogus* mosquitoes, are also regularly detected in populations living near rural and forest areas [25,26].

Although living conditions are substantially more precarious than those of mainland France, French and European status gives French Guiana higher healthcare, diagnosis and surveillance capacities than most South American countries.

Current epidemiological surveillance systems rely on data from general practitioners and hospitals. Laboratories assist in the early detection as well as in the spatial and temporal monitoring of epidemics [15]. However, these systems are not designed to estimate the actual impact of transmission. Indeed, arboviral diseases cause a wide clinical polymorphism ranging from asymptomatic forms to severe undifferentiated fevers that may lead to erroneous classifications. In addition, the problem of cross-reactivity of serological tests, resulting from the co-circulation of several arboviruses belonging to the same family, often implies multiple diagnostic tests and thus increases the overall cost, time and labor in areas where resources are sometimes limited [27].

It is essential to estimate the level of circulation of emerging arboviruses in French Guiana in order to understand the modalities of disease transmission, quantify the risk of future epidemics and ascertain the appropriate spatial scale for the deployment of control measures. Despite the health, social and economic impact of these arboviruses in French Guiana, very little is known about the immune status of populations and estimating the real impact of transmission is a major public health issue. Seroprevalence studies that quantify the proportion of the population with antibodies against epidemic and endemic arboviruses can help address such a knowledge gap in the level of circulation and spatial extent and support risk assessment of this emerging pathogen [28,29,30]. Previous efforts to understand population immunity to arboviruses have considered viruses individually. This means that the extent to which infection burden is shared between individuals from the same community or between communities is largely unknown. By considering multiple arboviruses within the same study, we can consider multiple pathogens simultaneously.

In this context, we conducted a large multiplexed serological survey to provide a comprehensive overview of the burden of DENV, CHIKV, ZIKV and MAYV in French Guiana, with useful findings for risk mapping, future prevention and control programs.

## 2. Materials and Methods

### 2.1. Study Design and Participants

A cross-sectional population-based serological survey and household interviews were conducted in French Guiana between June and October 2017. Data were collected through a standardized questionnaire installed on tablets to register demographics, socioeconomics and household characteristics.

We reproduce here details on the random household selection, sampling weights, interviews, and ethical considerations that were already described in Flamand et al. [31]. The French Guianese territory is composed of 22 municipalities that we broke down into seven geographical areas for statistical analysis.

A total of 1600 households were randomly selected for possible participation in the study from household databases maintained by the geographic information and knowledge dissemination unit of the Regional environment, planning and housing agency and the National Institute of Economic and Statistical Information (INSEE) [32]. A stratified simple random sampling was adopted to select households allowing an overrepresentation of the isolated and small municipalities. The global sampling fraction of the households was 1:49 varying from 1:103 to 1:5 according to the municipality. Details of the study design have been presented in Flamand et al. [31].

We applied a post-stratification adjustment to each of these weights to arrive at the final statistical weight for each subject. This adjustment helped us to weight the age–sex groups within each municipality to match the distribution in the French Guiana total population.

### 2.2. Ethical Consideration

The study was recorded on ClinicalTrials.gov (NCT03210363) and approved by the Sud-Ouest & Outre-Mer IV Ethical Research Committee (number CPP17-007a/2017-A00514-49) and by the French Data Protection Authority (number DR-2017–324) responsible for ethical issues and protection of individual data collection.

### 2.3. Blood Sample Collection

Blood samples were collected into 5 mL gold BD Vacutainer SST II advance tubes with gel for serum separation (BD Diagnostics, Le Pont de Claix, France). Immediately after puncture, samples were stored at 4–8 °C until centrifugation within 12 h. Sera were then frozen and stored at −20 °C until use at the National Reference Center for arboviruses in the Institut Pasteur in French Guiana.

### 2.4. Serological Diagnosis

All serum samples were tested for immunoglobulin G (IgG) antibodies against DENV, CHIKV, ZIKV and MAYV using a recombinant antigen-based microsphere immunoassay (MIA) adapted from Beck et al. [33].

This MIA was based on a panel of recombinant viral proteins corresponding to the domain III of the envelope glycoprotein for flaviviruses (DENV 1 to 4 and ZIKV), shown to limit cross-reactivity between flaviviruses, and to the glycoprotein E2 for alphaviruses (CHIKV and MAYV), whereas a recombinant human protein (O^6^-methylguanine DNA methyltransferase) was used as control antigen

Distinct MagPlex microsphere sets (Luminex Corp., Austin, TX, USA) were, respectively, bound to viral and control proteins using the Amine Coupling Kit (Bio-Rad Laboratories, Hercules, CA, USA) according to manufacturers’ instructions. The MIA procedure was performed as described previously with minor modifications [34]. Briefly, microsphere mixtures were sequentially incubated in the dark under constant shaking with a 1:400 dilution of serum samples and 2 μg/mL anti-human IgG phycoerythrin-conjugated antibody (Jackson Immunoresearch, West Grove, PA, USA). After the final incubation, the median fluorescence intensity (MFI) of each microsphere set was quantified using a MAGPIX instrument (Luminex Corp., Austin, TX, USA). For each sample, DENV 1 to 4, ZIKV, CHIKV and MAYV, relative fluorescence intensities (RFI) were calculated by dividing the MFI signal measured for each type of microsphere set by the MFI signal obtained for the control microsphere set.

Microneutralization (MNT) tests were performed to improve the interpretation of DENV, CHIKV, ZIKV and MAYV RFI and to determine MIA cut-offs based on sensitivity, specificity and accuracy between MIA and MNT. Briefly MNTs were conducted in serial 2-fold dilutions of heat inactivated sera starting at 1:10 mixed in equal volume with 100 tissue culture infectious dose 50 (TCID 50) of DENV 1 to 4, ZIKV, MAYV or CHIKV (French Guiana strains). After incubation at 37 °C for 1 h, mixtures were transferred onto 96-well tissue culture plates containing subconfluent Vero cells. The neutralization titer was expressed as the reciprocal of the highest serum dilution at which infection is blocked. A serum was considered positive for titer above 20.

A total of 422 sera were selected according to a municipality-stratified simple random sampling method to test DENV-1 to DENV-4 MNT. A sample was considered DENV positive if MIA ratio cut-offs for DENV-1 were greater than or equal to 1.5, or if its ratio for DENV-2 was greater than or equal to 1.74, or if its ratio for DENV-3 was greater than or equal to 2.1, or if its ratio for DENV-4 was greater than or equal to 1.74. Using the results from the MNTs as the gold standard, the obtained classification indicated a sensitivity of 95% and a specificity of 91%.

For ZIKV, 235 first samples were selected to evaluate the correlation between ZIKV MIA and MNT and to determine MIA cutoffs. A sample was considered ZIKV positive if its MIA ratio was >2.5 and negative for a value < 1.5. All samples with an MIA ratio between 1.5 and 2.5 were tested by MNT and considered positive for neutralizing titers >20. A total of 607 sera were tested by anti-ZIKV MNTs. MNT was also systematically performed where the MIA ratio was <1.5 for anyone who had reported an arboviral-like infection in the last 2 years. Methodological details have been described in Flamand et al. [31].

Finally, an analytical framework was developed to assess the extent of cross-reactivity between MAYV and CHIKV, and to determine the serological status of individuals based on a model-based classification that derives the probability of infection with MAYV and/or CHIKV for each possible value of the assay. A total of 100 sera were randomly selected for testing anti-MAYV and anti-CHIKV MNTs to validate the model-based classification. Using the results from the MNTs, the obtained classification indicated a sensitivity of 87% and a specificity of 94% for MAYV and a sensitivity of 100% and a specificity of 95% for CHIKV. Details of the model-based classification are described in Hozé et al. [35].

### 2.5. Statistical and Spatial Analyses

Weighted seroprevalence estimates were calculated, and associated factors were identified by using survey-weighted Poisson regression and prevalence ratios (PRs). The strength of association of the selected variables and DENV, CHIKV, ZIKV and MAYV seropositivity were estimated by crude and adjusted PRs with their 95% confidence interval (CI), all confidence intervals excluding 1.0 being considered as significant.

The inverse distance weighting (IDW) interpolation method was used to represent the spatial distribution of seroprevalence across the country. Choropleth maps were made to represent the spatial distribution of arbovirus transmission risk by comparing, in pairs, seroprevalence levels of the different viruses [36]. Seroprevalence ratios of each of the represented viruses were categorized into four discrete classes to use unique sixteen-color grids on the developed maps. Urbanization level was obtained from a land use classification based on the proportion of households within a 1km buffer (Rural: *p* < 50%; Urban: *p* ≥ 50%). Statistical analyses were carried out using survey capabilities of Stata version 15 statistical software [37]. French Guiana’s layers were drawn using geodata from OpenStreetMaps (http://www.openstreetmap.org, accessed on 1 February 2021) and spatial analyses were performed using QGIS 2.18 software [38].

## 3. Results

### 3.1. Overall Seroprevalence of Emerging Arboviruses

In total, 1415 households and 2697 individuals were included between June and October 2017 from the 22 municipalities of French Guiana (Table 1).

The mean household size was 1.9 individuals [range: 1 to 11]. The mean age was 34.1, ranging from 2 to 75 years old. Comparison of the socio-demographic characteristics of the study sample to the census data demonstrated an over-representation of women (58.9% vs. 50% in the general population of French Guiana) and adults over 25 years (64% vs. 53% in French Guiana). These differences were accounted for in the analyses of seroprevalence and risk factors by allocating post-stratification weight to each participant.

**Figure 2:**
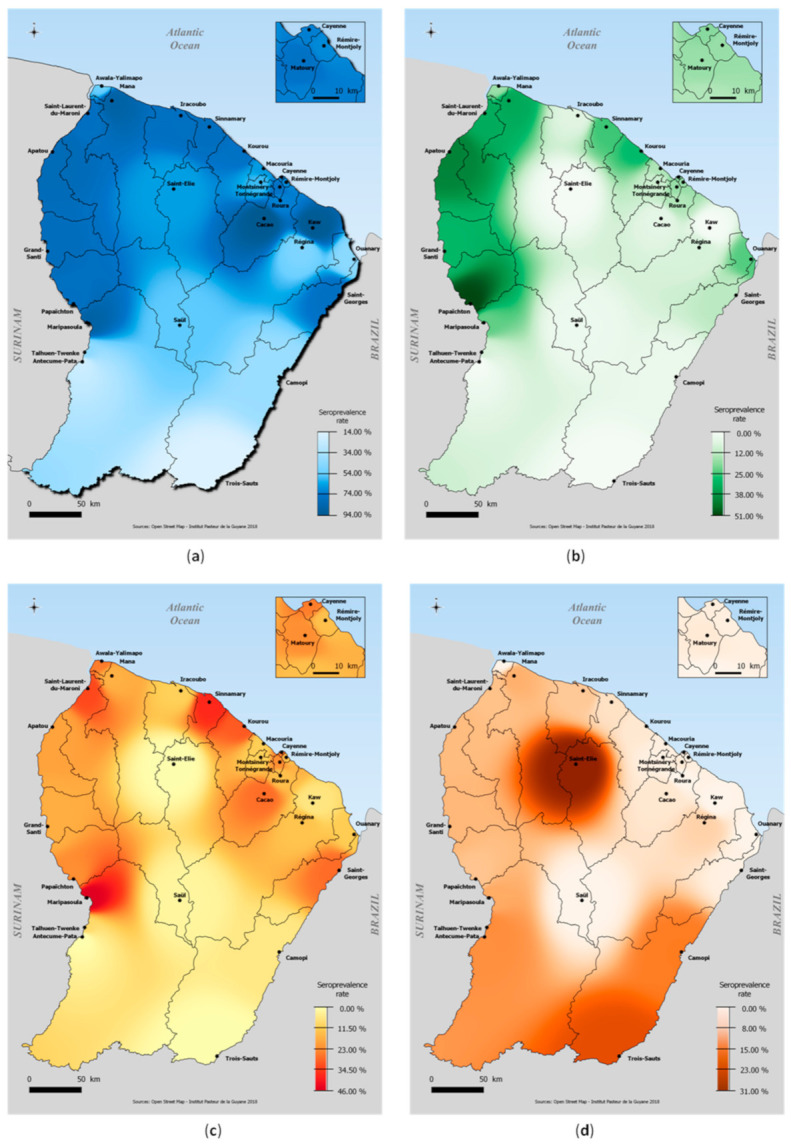
Spatial distribution of (**a**) DENV seroprevalence, (**b**) CHIKV seroprevalence, (**c**) ZIKV seroprevalence and (**d**) MAYV seroprevalence, French Guiana, 2017.

Burden and spatial distributions of DENV, CHIKV and ZIKV are presented in Table 1 and Figure 2.

The overall weighted DENV seroprevalence was estimated at 73.1% (70.6–75.4) in the whole territory with estimations by serotype at 68.9% (66.0–71.2) for DENV-1, 38.8% (36.2–41.4) for DENV-2, 42.3% (39.7–44.9) for DENV-3, and 56.1% (53.3–58.7) for DENV-4. The proportion of individuals who were positive for only one of the dengue serotypes was 16.0% (14.3–18.0), while 57.0% (54.3–59.8) of individuals were positive for two or more serotypes.

The overall weighted seroprevalence of CHIKV, ZIKV and MAYV antibodies was 20.3% (17.7–23.1), 23.3% (20.9–25.9) and 3.3% (2.7–4.1), respectively.

**Figure 3:**
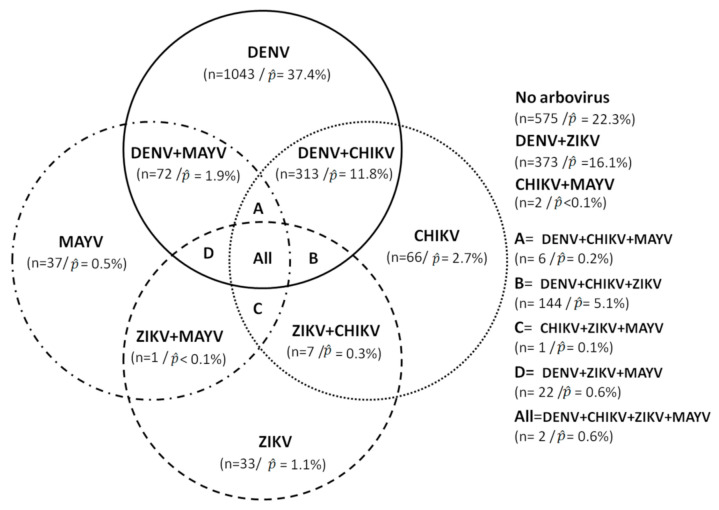
Sample distribution of arboviruses infections and co-infections.

Population estimates indicated that 22.3% of the population was negative for all four arboviruses, 37.4% were positive for only DENV, 16.1% for DENV and ZIKV, 11.8% for DENV and CHIKV, 5.1% for DENV, CHIKV, and ZIKV, 0.05% with CHIKV and MAYV, and 0.06% were positive for all arboviruses (Figure 3).

### 3.2. Spatial Distribution and Factors Associated with DENV, CHIKV and ZIKV

Relatively homogeneously throughout the territory, DENV seroprevalence levels were between 70% and 80% over a large majority of the geographic areas. The highest DENV infection risks were observed in the Low Maroni area (78.6% (73.4–83.0)), the Low Oyapock area, (76.4% (62.8–86.2)) and the coastal area (72.5% (9.5–75.2)). Five communities had seroprevalence levels above 80% including small villages of Cacao and Kaw (94.8% (82.2–98.6) and 93.5% (63.6–99.2)), Maripasoula center area (87.9% (75.2–94.5)), Mana municipality (87.9% (78.1–93.7)) and Apatou municipality (81.5% (68.1–90.1)).

The regions of High Oyapock and High Maroni, located in the southern part of the Amazon forest area, had the lowest seroprevalence levels and the most isolated villages of Trois-Sauts (13.9% (7.0–25.7)) and Antécume Pata (20.8% (10.3–37.5)) appeared to have been minimally affected by DENV circulation (Table 1).

While CHIKV seroprevalence was highest in the western municipalities along the Low Maroni villages, very low levels were estimated in the interior area and no seropositive individuals were identified in the village of Kaw, Antecume-Pata and in Saint-Elie (Table 1).

Similarly, while ZIKV circulated in a large part of French Guiana, it barely reached the interior and remote villages located in the most isolated forest areas (Saint-Elie, Saül, Talhuen-Twenke villages, and Camopi) (Table 1). High probabilities of infection were observed in the main population centers along the Maroni river, in the coastal area (Kourou: 30.1% (23.8–37.3); Cayenne: 25.2% (20.2–30.9)), and in Low-Oyapock (Saint-Georges: 27.6% (13.6–47.9)). Two smaller geographical areas in the coastal area were also strongly impacted (Sinnamary: 37.4% (24.0–53.1); Cacao: 27.3% (12.2%–50.4%)) (Table 1).

**Figure 4:**
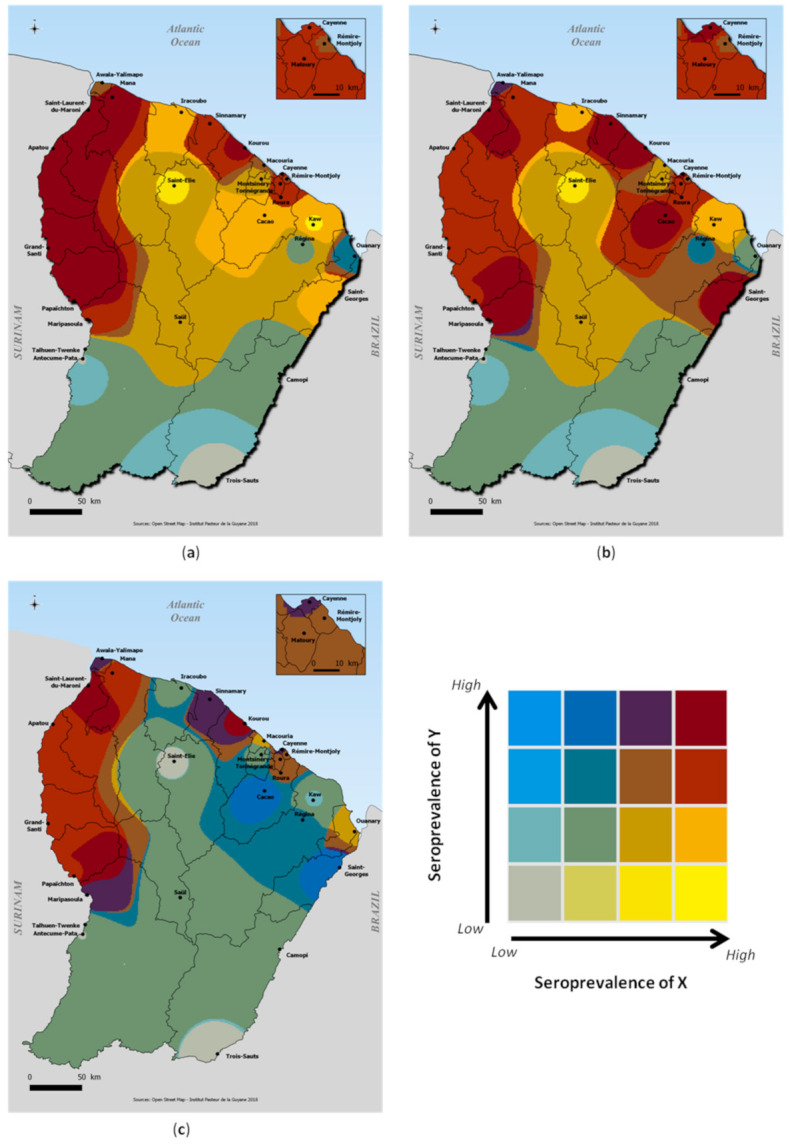
Graphical representation of the correlations between DENV, CHIKV and ZIKV seroprevalences levels; (**a**) DENV (X) vs. CHIKV (Y); (**b**) DENV (X) vs. ZIKV (Y); (**c**) CHIKV (X) vs. ZIKV seroprevalence (Y).

Living in the western part of the territory, along the Maroni river and in an urban area was significantly associated with being seropositive in both univariate and bivariate multivariate analyses for DENV, CHIKV and ZIKV (Table 2). Analysis of the choropleth maps (Figure 4) shows that, overall, and apart from the most important population basins in the urbanized coastal zone, the western zone of French Guiana, along the Maroni River, from Maripasoula to Mana, represent a geographic area with a high risk of transmission of DENV, CHIKV and ZIKV. In contrast, the interior zone was associated with relatively high levels of DENV but low levels of CHIKV and ZIKV that have been responsible for single epidemic emergences. The High Oyapock and High Maroni areas remain little exposed to the risk of transmission of these three arboviruses.

Benefiting from universal health coverage or state medical assistance and having a low family income were associated with seropositivity at the univariate level for all three urban arboviruses. However, these socioeconomic factors were no longer significant in multivariate analyses for ZIKV. Women were at higher risk for CHIKV, while sex did not appear in multivariate analysis for DENV and ZIKV. For DENV, the risk of past infection was significantly higher for people over 65 years.

### 3.3. Spatial Distribution and Factors Associated with MAYV Infection

Spatial distribution of seroprevalence levels is shown in Figure 2. Highest seroprevalences were observed in the sparsely populated high Oyapock/Interior region. The highest seroprevalence was observed in Saint-Elie (30.5% (9.3–65.3)) and in the isolated village of Trois-Sauts (23.5 (12.1–40.7)). Lower infections risks were observed in the coastal and urban area (Montsinnery-Tonnegrande: 0.9% (0.1–6.1); Remire-Montjoly: 1.0% (0.2–4.2); Roura: 1.0% (0.1–6.1)). There were no cases in several municipalities (Awala, Ouanary and Saül). Age was positively associated with being seropositive for MAYV in both univariate and multivariate (Table 3). In addition, being male, not having general social coverage and living along the Maroni River, in the Interior or in the High Oyapock were risk factors for MAYV infection. Although in the univariate model, the rural area was significantly more at risk of transmission than the urban area, the difference in risk was not significant in the multivariate model, particularly when geographic area was included in the model.

## 4. Discussion

As the only continental territory of France and the European Union in South America, French Guiana has been strongly affected by re-emerging and emerging arbovirus epidemics in recent years. In the absence of a vaccine, prevention and control strategies are limited to vector control actions, which remain extremely difficult to direct in the context of arbovirus epidemics. In French Guiana, as in most of the affected areas, vector control actions are carried out in and around the homes of confirmed cases reported by the surveillance systems [15].

While this strategy has the advantage of limiting the infection of vectors that may be present in the homes of cases, provided that the actions are carried out within a reasonable time frame, it does not always allow the targeting of areas where the risk of transmission is greatest. However, in territories where resources are particularly limited, the orientation of actions towards priority areas represents a major challenge in a context of active virus circulation.

By allowing us to describe the common experiences of infection by the main arboviruses that have emerged in French Guiana, this large multiplexed serological survey allows us to estimate the shared burden of infection between individuals in the different communities and to identify, consequently, the areas to be targeted in order to implement prevention and response strategies in an optimal way in case of a future epidemic.

The results presented here allow us to assess the impact of the main emerging arboviruses and to better understand their transmission modalities at the scale of French Guiana.

DENV seroprevalence was estimated at 73.1% in the whole territory with specific risks of past infection at 68.9% for DENV-1, 38.8% for DENV-2, 42.3% for DENV-3, and 56.1% for DENV-4.

These findings were consistent with epidemiological knowledge obtained from dengue surveillance data since all dengue virus serotypes have been identified in French Guiana during previous and recent epidemics. Ten major epidemics have been identified between 1992 and 2013 [14]. All four DENV serotypes have been circulating in French Guiana in the last 20 years, with changes in predominant serotypes that have varied over time concomitant with reported epidemics [15,20,21].

However, although the use of defined thresholds for each serotype allows the classification of DENV serostatus for all serotypes, it is likely that the specific prevalence of some serotypes, including DENV2, which has caused 5 major epidemics in the past 20 years [14,15,20,21], is underestimated because of a lack of sensitivity of the DENV-2 MIA or a weaker immune response, as described previously [29]. In addition, comparison of MIA and MNT results highlighted limitations of the MIA test in determining serological status relative to a DENV serotype-specific and therefore in providing seroprevalence estimates for DENV serotype-specifics. Further work should be conducted to identify the best approaches to study the impact of different DENV serotypes in different communities in French Guiana.

While nearly three-quarters of the population has already been infected with one of the dengue viruses, nearly a quarter of the population has been infected with chikungunya and Zika viruses.

These results show that following the recent epidemic emergence of two arboviruses such as CHIKV and ZIKV, the majority of the population remains susceptible, which could potentially facilitate the risk of further re-emergences.

Our results also revealed significant heterogeneity in the risk of infection with these two viruses, with seroprevalence levels of about 40% in some communities on the coastal area or “Low Maroni”, but low transmission risks in the central part of the territory and in the most isolated river areas. This distribution contrasts somewhat with that of DENV, which has been marked by several successive epidemics resulting in higher transmission risks in most geographic areas, including the central and interior zone. Only the most isolated villages of the High Maroni and High Oyapock had concomitantly low levels of DENV, CHIKV and ZIKV.

The observed differences in infection risks across the territory appear to reflect, in part, differences in the distribution of mosquitoes in the territory. Population movement, economic development, and urbanization have facilitated the geographic expansion of *Ae. aegypti* and its establishment in almost all inhabited areas of French Guiana, even in villages along the Maroni River to the main area of Maripasoula [39,40]. However, to date, no studies have reported the presence of *Ae. aegypti* populations in the most remote villages, including Antecume Pata, Twenke-Talhuen, and Camopi [40], where seroprevalence ratios range from 0 to 8%. Furthermore, despite the strengthening of existing epidemiological surveillance systems [15] and entomo-epidemiological surveys coordinated by local health authorities when a clinical or confirmed case appeared in these areas, no autochthonous transmission of Aedes-borne diseases was identified in these villages. Surprisingly, the risk of infection from viruses transmitted by *Ae. aegypti*, which is well known to be a preferentially urban vector, was very high in the communes or villages along the Maroni River in a much less urbanized environment than the coastal area. Seroprevalence levels were often higher than those observed in the coastal zone. This situation probably reflects a socio-economic and environmental context favorable to the transmission of arboviruses due to explosive demographic growth accentuated by large movements of people living in precarious conditions [41] and high anthropization conducive to the proliferation of *Ae. aegypti* populations in this part of the territory. Although entomological data on vector population densities that could confirm this hypothesis are not available, previous work has already demonstrated the geographic expansion of *Ae. aegypti* and its establishment in almost all inhabited areas of French Guiana, even in villages along the Maroni River to the main area of Maripasoula [39,40]. Low logistical and human resources for vector control activities may also have contributed to higher transmission intensities in this part of the territory. Indeed, the surveillance and management program for arboviral diseases in French Guiana includes the reduction of *Ae. aegypti* density throughout the year, which is intensified during epidemics. These activities include indoor and outdoor space spraying of deltamethrin against adults and elimination of breeding sites or their treatment with *Bacillus thuringiensis* var. *israelensis* larvicides [42]. Although in epidemic contexts, routine activities are strengthened in areas where the number of cases is high, it is common that the deployment of control activities is limited in this more difficult-to-access area. Finally, it cannot be ruled out that people from this area of the country are less willing to go for screening, as a result of cultural, social and/or behavioural particularities, limiting the early outbreak detection and consequently the timely deployment of control measures.

The study of factors associated with the risk of infection confirmed the impact of low socio-economic levels, urban area for *Ae. aegypti*-transmitted viruses and rural area for Mayaro virus risk. Consistently, the risks of infection of endemic diseases such as DENV and MAYV were age-dependent, unlike the risks of infection related to the recent emergences of CHIKV and ZIKV, which were not related to age-dependent exposure duration. With the exception of the risk of infection for MAYV, which was higher in men, there was no gender difference in infection risks for DENV, CHIKV, and ZIKV.

Epidemiological interpretation of serological results is often compromised by cross-reactivity between circulating pathogens [30]. Our study showed the importance of adapting and combining different approaches to assess, in an important context of co-circulation, the immune status of the population according to the results of the diagnostic tests used.

While the MNT results showed that DENV serological status could be determined from the MIA intensity ratios of the different dengue serotypes, it was necessary to adapt the strategy to define the ZIKV serological status from the MIA ratio by systematically performing MNT tests when the MIA ratio was between certain values that had been previously defined [31].

Regarding the determination of the serological status for MAYV and CHIKV, we had to combine the serological results with modeling techniques by integrating several data streams to determine the serological status for MAYV, which was strongly impacted by cross-reactions with CHIKV [35].

We identified only eleven individuals with evidence of historical infections with both CHIKV and MAYV alphaviruses. While this may indicate cross-immunity between the two alphaviruses, it could more likely be due to the fact that the vectors occupy different ecological niches. This hypothesis is also supported by a low number of individuals who were positive for both ZIKV and MAYV (*n* = 26), which belong to different viral families.

The number of co-infections between DENV and ZIKV (*n* = 541, 21.8% [19.4–24.3]) was comparable to the number of co-infections between DENV and CHIKV (*n* = 465, 23.5% [20.7–26.5]), most likely reflecting a risk of environmental exposure to the different viruses transmitted by *Ae. aegypti*.

Herein, we provide a consistent overview of the burden and spatial distribution of major emerging arboviruses in French Guiana. Given the large proportion of clinically asymptomatic infections and that the disease is largely underreported, our results provide distinct and useful information by geographic area and population subgroups in a continental area frequently exposed to arboviruses.

Our results also underscore the need to strengthen MAYV surveillance in the region to be able to rapidly detect any substantial changes in MAYV circulation patterns that may indicate increasing emergence.

## Figures and Tables

**Table 1:** Description of the household selection process and weighted seroprevalence estimated by municipality.

Municipality (i) Sub-Municipalities Areas	Population	No. of Households	No.(%) of Enrolled Households	No. of Enrolled Individuals	DENV Weighted Seroprevalence (95% CI)	CHIKV Weighted Seroprevalence (95% CI)	Zika Weighted Seroprevalence (95% CI)	Mayaro Weighted Seroprevalence (95% CI)
Cayenne	57,614	21,659	196 (0.9%)	446	72.0 (66.7–76.7)	18.1 (13.0–24.7)	25.2 (20.2–30.9)	1.4 (0.6–3.0)
Matoury	32,427	10,778	136 (1.3%)	265	76.8 (69.7–82.6)	16.2 (11.0–23.1)	22.7 (16.5–30.5)	2.0 (0.9–4.4)
Saint-Laurent	43,600	9770	170 (1.7%)	301	78.0 (71.7–83.2)	32.1 (24.2–41.2)	32.4 (25.7–39.9)	9.1 (6.2–13.0)
Kourou	26,221	8205	167 (2.0%)	294	77.0 (70.2–82.6)	28.3 [21.8–35.8)	30.1 (23.8–37.3)	2.3 (1.1–4.9)
Remire-Montjoly	23,976	8117	105 (1.3%)	192	69.9 (61.3–77.2)	16.2 (9.7–26.0)	13.7 (8.5–20.9)	1.0 (0.2–4.2)
Macouria	11,719	4218	75 (1.8%)	164	66.0 (56.8–74.2)	13.8 (7.8–23.3)	10.9 (6.8–17.1)	1.1 (0.3–4.6)
Mana	10,241	2297	74 (3.2%)	96	87.9 (78.1–93.7)	33.0 (23.3–44.4)	16.7 (10.6–25.4)	11.5 (5.9–21.2)
Maripasoula	11,856	1955	74 (3.8%)	145	62.3 (51.2–72.3)	13.7 (7.9–22.6)	26.7 (16.6–40.0)	13.6 (8.7–20.7)
*« Maripasoula center area »*	.	.	*50*	*77*	87.9 (75.2–94.5)	22.3 (12.4–36–7)	45.6 (30.0–62.0)	12.2 (5.9–23.3)
*“Twenke-Talhuen” village*	.	.	*14*	*33*	43.4 (32.1–55.3)	6.9 (1.8–23.5)	8.4 (2.6–24.0)	15.5 (7.8–28.4)
*« Antecume-Pata » village*	.	.	*10*	*35*	20.8 (10.3–37.5)	0	0	15.1 (6.1–32.8)
Apatou	8431	1839	45 (2.5%)	62	81.5 (68.1–90.1)	40.5 (29.2–52.9)	19.1 (10.2–32.9)	10.0 (4.7–20.3)
Grand-Santi	6969	1447	28 (1.9%)	61	79.6 (65.4–88.9)	29.1 (17.6–44.0)	17.1 (8.3–32.0)	10.6 (4.8–21.7)
Saint-Georges	4020	1208	32 (2.7%)	86	77.6 (63.4–87.4)	12.1 (4.2–30.3)	27.6 (13.6–47.9)	0.8 (0.1–5.3)
Papaïchton	7266	1150	32 (2.8%)	49	78.0 (52.9–91.8)	50.3 (34.2–66.3)	22.7 (11.1–40.8)	8.5 (3.4–19.8)
Sinnamary	2957	1092	30 (2.8%)	39	79.1 (60.2–90.4)	25.0 (10.2–49.5)	37.4 (24.0–53.1)	3.6 (0.5–22.0)
Roura	3713	983	39 (4.0%)	70	80.2 (68.2–88.5)	9.7 (4.2–20.8)	18.7 (9.7–32.9)	1.0 (0.1–6.1)
*“Roura main area”*	.	.	*26*	*45*	71.9 (57.3–83.0)	13.3 (5.3–29.5)	13.7 (5.1–31.7)	0
*“Cacao village”*	.	.	*13*	*25*	94.8 (82.2–98.6)	3.4 (0.5–19.5)	27.3 (12.2–50.4)	2.6 (0.5–13.3)
Montsinnery–Tonnegrande	2473	898	29 (3.2%)	66	59.0 (45.2–71.5)	8.6 (2.2–28.5)	10.7 (2.7–33.9)	0.9 (0.1–6.1)
Iracoubo	1878	585	29 (5.0%)	53	78.1 (65.1–87.2)	4.7 (0.6–28.1)	10.7 (4.8–22.1)	7.9 (2.6–22.1)
Regina	946	401	43(10.7%)	75	54.8 (42.6–66.5)	5.4 (2.1–13.4)	12.0 (5.8–23.4)	5.2 (2.0–13.1)
*“Regina main area”*	.	.	*33*	*64*	48.4 (36.6–60.3)	6.3 (2.4–15.6)	13.1 (5.9–26.3)	6.1 (2.3–15.4)
*“Kaw village”*	.	.	*10*	*11*	93.5 (63.6–99.2)	0	5.9 (0.8–34.0)	0
Camopi	1769	346	50 (14.5%)	115	31.8 (22.7–42.6)	1.6 (0.4–6.7)	3.7 (1.3–9.8)	19.2 (13.2–27.0)
*“Camopi main area”*	.	.	*34*	*83*	39.2 (27.7–52.1)	2.3 (0.5–9.5)	5.3 (2.0–13.5)	17.4 (11.0–26.5)
*“Trois-Sauts village”*	.	.	*16*	*32*	13.9 (7.0–25.7)	0	0	23.5 (12.1–40.7)
Awala	1379	330	28 (8.5%)	60	54.3 (35.7–71.8)	16.0 (7.5–31.1)	25.5 (10.2–50.7)	0
Saint-Elie	95	143	10 (7.0%)	11	65.0 (32.6–87.7)	0	0	30.5 (9.3–65.3)
Ouanary	165	140	5 (3.6%)	13	44.2 (11.8–82.4)	23.3 (4.7–64.9)	11.3 (3.0–34.5)	0
Saül	150	94	18 (19.2%)	34	55.4 (40.0–69.8)	1.8 (0.2–13.3)	2.1 (0.3–15.3)	0
Total	259,865	77,655	1415	2697	73.1 (70.6–75.4)	20.3 (17.7–23.1)	23.3 (20.9–25.9)	3.3 (2.7–4.1)

**Table 2:** Factors associated with DENV, CHIKV and ZIKV seropositivity.

Characteristic	DENV			CHIKV			ZIKV		
	Weighted Prevalence %	Univariate	Multivariate	Weighted Prevalence %	Univariate	Multivariate	Weighted Prevalence %	Univariate	Multivariate
	(95% CI)	PR (95% CI)	PR (95% CI)	(95% CI)	PR (95% CI)	PR (95% CI)	(95% CI)	PR (95% CI)	PR (95% CI)
Sex									
Male	72.2 (68.7–75.5)	Ref	Ref	18.5 (15.5–22.0)	Ref	Ref	22.8 (19.5–26.5)	Ref	Ref
Female	73.9 (70.8–76.7)	1.0 (1.0–1.1)	1.0 (0.9–1.0)	22.0 (19.0–25.2)	1.2 (1.0–1.4) *	1.1 (1..0–1.3)	23.8 (21.0–26.8)	1.0 (0.9–1.2)	1.0 (0.8–1.2)
Age, y									
2–14	52.8 (47.1–58.3)	Ref	Ref	18.0 (13.2–24.0)	Ref	Ref	24.7 (19.8–30.3)	Ref	Ref
15–24	76.9 (71.6–81.4)	1.5 (1.3–1.6) ***	1.5 (1.3–1.7) ***	24.7 (19.9–30.3)	1.4 (1.0–1.9)	1.3 (0.9–1.7)	25.9 (21.0–31.6)	1.0 (0.8–1.4)	1.0 (0.8–1.4)
25–34	77.6 (72.7–81.8)	1.5 (1.3–1.6) ***	1.6 (1.4–1.8) ***	23.1 (18.5–28.4)	1.3 (0.9–1.7)	1.2 (0.9–1.6)	23.6 (19.2–28.7)	0.9 (0.7–1.3)	1.0 (0.7–1.3)
35–44	79.1 (73.6–83.8)	1.5 (1.3–1.7) ***	1.7 (1.5–1.9) ***	20.3 (16.0–25.4)	1.1 (0.8–1.6)	1.1 (0.8–1.5)	19.7 (15.8–24.3)	0.8 (0.6–1.1)	0.8 (0.6–1.1)
45–54	86.2 (81.3–89.8)	1.6 (1.4–1.8) ***	1.8 (1.6–2.0) ***	15.7 (12.0–20.4)	0.9 (0.6–1.3)	0.8 (0.6–1.1)	21.2 (16.5–26.8)	0.8 (0.6–1.2)	0.9 (0.6–1.2)
55–64	85.6 (80.1–89.8)	1.6 (1.4–1.8) ***	1.7 (1.5–1.9) ***	16.0 (11.5–21.9)	0.9 (0.6–1.4)	0.9 (0.6–1.3)	18.9 (14.3–24.6)	0.8 (0.5–1.1)	0.8 (0.5–1.1)
≧65	90.5 (82.9–95.0)	1.7 (1.5–1.9) ***	1.8 (1.6–2.0) ***	23.0 (18.0–35.8)	1.4 (0.9–2.3)	1.3 (0.8–2.0)	28.3 (20.4–37.7)	1.1 (0.8–1.7)	1.2 (0.8–1.7)
Birth place									
French Guiana	71.5 (68.1–74.7)	Ref	Ref	17.2 (13.8–21.2)	Ref	Ref	23.4 (20.2–27.1)	Ref	Ref
Surinam	83.9 (75.8–89.6)	1.2 (1.1–1.3) ***	1.0 (0.9–1.1)	32.6 (25.2–41.0)	1.9 (1.4–2.6) ***	1.4 (1.0–1.9) *	27.0 (20.2–35.1)	1.1 (0.8–1.6)	1.1 (0.8–1.6)
Brazil	76.3 (67.6–83.3)	1.1 (0.9–1.2)	0.9 (0.8–1.0) *	7.8 (4.1–14.2)	0.4 (0.2–0.9) *	0.4 (0.2–0.8) *	19.1 (13.3–26.7)	0.8 (0.6–1.2)	0.8 (0.6–1.3)
Other South America	88.9 (69.6–96.6)	1.2 (1.1–1.4) ***	1.0 (0.9–1.1)	31.9 (19.9–46.9)	1.8 (1.1–3.0) **	1.7 (1.1–2.6) *	27.6 (17.7–40.2)	1.2 (0.8–1.8)	1.2 (0.7–1.9)
Haïti	95.1 (90.3–97.6)	1.3 (1.2–1.4) ***	1.1 (1.0–1.2) *	48.9 (41.2–56.6)	2.8 (2.2–3.7) ***	2.2 (1.6–3.2) ***	34.3 (27.2–42.3)	1.5 (1.1–1.9) **	1.3 (0.9–1.8)
Caribbean island	94.6 (88.6–97.5)	1.3 (1.2–1.4) ***	1.1 (1.1–1.2) ***	25.3 (17.1–35.8)	1.5 (1.0–2.2)	1.9 (1.3–2.9) ***	20.6 (13.5–30.2)	0.9 (0.6–1.3)	0.9 (0.6–1.5)
Europe	46.7 (40.8–52.7)	0.7 (0.6–0.8) ***	0.6 (0.5–0.7) ***	6.9 (4.2–11.3)	0.4 (0.2–0.7) ***	0.6 (0.3–1.1)	17.0 (12.4–22.8)	0.7 (0.5–1.0)	0.8 (0.6–1.2)
Asia	70.9 (43.3–88.6)	1.0 (0.7–1.4)	0.8 (0.6–1.1)	15.6 (4.4–42.6)	0.9 (0.3–3.0)	1.4 (0.4–5.2)	4.6 (1.1–17.6)	0.2 (0.0–0.8) *	0.2 (0.1–1.0)
Africa	67.9 (43.3–85.5)	0.9 (0.7–1.3)	0.8 (0.6–1.1)	17.2 (5.3–43.5)	1.0 (0.3–3.0)	1.9 (0.6–5.8)	12.4 (3.1–38.6)	0.5 (0.1–2.0)	0.6 (0.2–2.5)
Others	18.0 (2.6–63.9)	0.3 (0.0–1.4)	0.2 (0.0–1.1)	31.7 (4.9–80.5)	1.8 (0.4–8.3)	2.4 (0.8–7.7)	6.1 (0.7–36.6)	0.3 (0.0–2.0)	0.2 (0.0–2.0)
Region of residence									
Coastal area	72.5 (69.5–75.2)	Ref	Ref	18.0 (15.1–21.3)	Ref	Ref	23.2 (19.4–25.3)	Ref	Ref
Low Maroni	78.6 (73.4–83.0)	1.1 (1.0–1.2)	1.1 (1.0–1.2) *	32.6 (26.2–39.8)	1.8 (1.4–2.4) ***	1.8 (1.2–2.7) **	28.8 (23.4–34.8)	1.3 (1.0–1.6) *	1.3 (1.0–1.7) *
High Maroni	67.2 (57.2–75.8)	0.9 (0.8–1.0)	0.9 (0.8–1.0)	25.0 (18.3–33.1)	1.4 (1.0–1.9)	1.9 (1.3–2.9) **	25.4 (17.2–36.0)	1.14 (0.8–1.7)	1.5 (1.0–2.4) *
Low Oyapock	76.4 (62.8–86.2)	1.0 (0.9–1.2)	1.2 (1.0–1.4) *	12.5 (4.6–29.7)	0.69 (0.3–1.8)	1.1 (0.4–3.0)	27.0 (13.5–9.8)	1.2 (0.6–2.3)	1.7 (0.9–3.4)
High Oyapock	31.8 (22.7–42.6)	0.4 (0.3–0.6) ***	0.4 (0.3–0.6) ***	1.6 (0.4–6.7)	0.1 (0.0–0.4) ***	0.1 (0.0–0.5) **	3.7 (1.3–6.9)	0.2 (0.1–0.5) ***	0.2 (0.1–0.6) **
Interior	60.6 (41.7–76.7)	0.8 (0.6–1.1)	0.8 (0.6–1.1)	0.8 (0.1–6.1)	0.04 (0.0–0.4) *	0.1 (0.0–0.7) *	1.0 (0.1–6.9)	0.04 (0.0–0.3) *	0.1 (0.0–0.5) *
Type of zone									
Rural	70.4 (66.7–73.8)	Ref	Ref	16.9 (14.0–20.2)	Ref	Ref	19.4 (16.2–23.1)	Ref	Ref
Urban	74.1 (70.9–76.9)	1.1 (1.0–1.2)	1.0 (1.0–1.1)	21.5 (18.3–25.2)	1.3 (1.0–1.6) *	1.4 (1.1–1.8) **	24.8 (21.7–28.1)	1.3 (1.0–1.6) *	1.5 (1.1–1.9) **
Household income, €									
<1000	79.9 (75.0–84.1)	Ref	Ref	31.8 (26.7–37.4)	Ref	Ref	26.9 (22.2–32.0)	Ref	Ref
1000–2999	73.2 (69.1–77.0)	0.9 (0.8–1.0) *	1.0 (0.9–1.1)	19.9 (14.7–26.4)	0.6 (0.4–0.9) **	0.9 (0.7–1.2)	24.5 (19.7–29.9)	0.9 (0.7–1.2)	1.0 (0.7–1.3)
3000–4999	63.8 (56.3–70.8)	0.8 (0.7–0.9) ***	0.9 (0.8–1.0)	8.1 (5.0–12.7)	0.2 (0.1–0.4) ***	0.5 (0.3–0.8) **	17.7 (12.5–24.3)	0.6 (0.4–1.0) *	0.8 (0.5–1.2)
≧5000	62.0 (52.3–70.9)	0.8 (0.6–0.9) **	0.9 (0.8–1.1)	8.2 (4.1–15.7)	0.2 (0.1–0.5) ***	0.5 (0.3–1.1)	15.7 (9.4–25.0)	0.6 (0.3–1.0) *	0.9 (0.7–1.2)
NP	75.2 (70.1–79.6)	0.9 (0.8–1.0)	1.0 (0.9–1.0)	21.1 (17.1–25.7)	0.7 (0.5–0.9) **	0.7 (0.5–1.0) *	24.0 (19.8–28.8)	0.9 (0.7–1.2)	1.26 (0.8–2.0)
Health insurance status									
General social coverage	69.1 (65.8–72.3)	Ref	Ref	11.9 (9.4–14.9)	Ref	Ref	20.2 (17.3–23.5)	Ref	Ref
Universal health coverage	75.6 (71.8–79.1)	1.1 (1.0–1.2) **	1.0 (0.9–1.1)	26.5 (22.1–31.4)	2.2 (1.7–3.0) ***	1.4 (0.9–2.1)	25.4 (21.5–29.8)	1.2 (1.0–1.6) *	1.0 (0.7–1.3)
State medical assistance	90.0 (81.6–94.9)	1.3 (1.2–1.4) ***	1.1 (1.0–1.2) *	51.0 (41.5–60.4)	4.3 (3.2–5.8) ***	1.8 (1.2–2.8) **	36.5 (27.5–46.6)	1.8 (1.3–2.5) ***	1.3 (0.9–1.9)

*** *p* < 0.001; ** *p* < 0.01; * *p* < 0.05; Ref = reference group (PR = 1).

**Table 3:** Factors associated with MAYV seropositivity.

Characteristic	Mayaro		
	Weighted Prevalence %	Univariate	Multivariate
	(95% CI)	PR (95% CI)	PR (95% CI)
Sex			
Male	3.8 [2.9–5.1]	Ref	Ref
Female	2.8 [2.1–3.8]	0.7 [0.5–1.1]	0.6 [0.4–1.0] *
Age, y			
2–17	0.3 [0.1–1.1]	Ref	Ref
18–34	3.0 [2.0–4.6]	11.3 [2.6–48.5] ***	13.2 [3.0–58.8] ***
35–44	4.4 [2.8–6.9]	16.5 [3.8–71.2] ***	26.0 [5.7–118.6] ***
45–54	7.3 [4.8–11.0]	27.4 [6.4–117.2] ***	37.1 [8.3–165.7] ***
55–64	6.0 [3.8–9.2]	22.4 [5.2–96.6] ***	44.1 [10.0–194.7] ***
≧65	9.2 [4.8–16.7]	34.4 [7.5–157.9] ***	75.2 [15.9–354.4] ***
Birth place			
French Guiana	3.5 [2.6–4.6]	Ref	Ref
Surinam	15.4 [10.6–22.0]	4.4 [2.8–7.0] ***	1.3 [0.7–2.2]
Brazil	3.5 [1.3–9.2]	0.9 [0.3–2.8]	0.6 [0.2–1.9]
Others	1.1 [0.5–2.3]	0.3 [0.1–0.7] **	0.2 [0.1–0.5] ***
Region of residence			
Coastal area	1.6 [1.1–2.4]	Ref	Ref
Low Maroni	9.4 [6.9–12.7]	5.7 [3.5–9.2] ***	2.7 [1.5–4.6] ***
High Maroni	12.0 [8.0–17.7]	7.3 [4.2–12.6] ***	3.3 [1.8–6.0] ***
Low Oyapock	0.7 [0.1–5.1]	0.4 [0.1–3.3]	0.4 [0.0–3.3]
High Oyapock	19.2 [13.2–27.0]	11.6 [6.9–19.6] ***	4.9 [2.7–8.9] ***
Interior	16.4 [5.3–40.4]	9.9 [3.3–30.0] ***	3.2 [1.0–10.6] *
Type of zone			
Rural	5.8 [4.5–7.5]	Ref	Ref
Urban	2.4 [1.7–3.3]	0.4 [0.3–0.6] ***	0.8 [0.5–1.3]
Household income, €			
<1000	4.2 [2.8–6.1]	Ref	Ref
1000–2999	2.4 [1.5–3.7]	0.6 [0.3–1.0]	1.3 [0.7–2.3]
3000–4999	1.2 [0.4–3.3]	0.3 [0.1–0.9] *	1.1 [0.3–3.8]
≧5000	2.2 [0.7–7.4]	0.5 [0.1–1.9]	3.3 [0.8–13.5]
NP	5.3 [3.9–7.3]	1.3 [0.8–2.1]	1.3 [0.8–2.0]
Health insurance status			
General social coverage	1.6 [1.0–2.5]	Ref	Ref
Universal health coverage	5.5 [4.3–7.0]	3.5 [2.1–6.0] ***	2.9 [1.6–5.5] ***
State medical assistance	3.2 [1.5–6.8]	2.0 [0.8–5.0]	3.7 [1.3–10.1] ***

*** *p* < 0.001; ** *p* < 0.01; * *p* < 0.05; Ref = reference group (PR = 1).

## Data Availability

Data are from the EPIARBO study belonging to Institut Pasteur (25-28 rue du Docteur Roux, 75724 Paris, CEDEX 15, France). Access to data is restricted for legal reasons according to the French CNIL recommendations (Commission Nationale Informatique et Libertes) that require specific authorizations to transfer health individual data from one center to another. The data may be made available after obtaining approval from the French regulatory authority: CNIL, Commission Nationale Informatique et Libertes (3 Place de Fontenoy TSA 80715, 75334 Paris Cedex 07, France. (Tel: +33 (0)153732200). Request for data transfer can be sent to Clinical Core of the Center for Translational Science of Institut Pasteur, Paris (Tel.: +33(0)140613874; Fax: +33(0)140613977; https://research.pasteur.fr/en/team/clinical-core, accessed on 1 February 2021).

## References

[1] Gould E., Pettersson J., Higgs S., Charrel R., De Lamballerie X. (2017). Emerging arboviruses: Why today?. One Health.

[2] Gould E., Coutard B., Malet H., Morin B., Jamal S., Weaver S., Gorbalenya A., Moureau G., Baronti C., Delogu I. (2010). Understanding the alphaviruses: Recent research on important emerging pathogens and progress towards their control. Antivir. Res..

[3] Pettersson J.H.-O., Eldholm V., Seligman S.J., Lundkvist Å., Falconar A.K., Gaunt M.W., Musso D., Nougairède A., Charrel R., Gould E.A. (2016). How Did Zika Virus Emerge in the Pacific Islands and Latin America?. MBio.

[4] Bhatt S., Gething P.W., Brady O.J., Messina J.P., Farlow A.W., Moyes C.L., Drake J.M., Brownstein J.S., Hoen A.G., Sankoh O. (2013). The global distribution and burden of dengue. Nat. Cell Biol..

[5] Kraemer M.U.G., Reiner R.C., Brady O.J., Messina J.P., Gilbert M., Pigott D.M., Yi D., Johnson K., Earl L., Marczak L.B. (2019). Past and future spread of the arbovirus vectors Aedes aegypti and Aedes albopictus. Nat. Microbiol..

[6] Gubler D.J. (2011). Emerging vector-borne flavivirus diseases: Are vaccines the solution?. Expert Rev. Vaccines.

[7] Nero C. (2008). Chikungunya, the Traveling Virus. Clin. Microbiol. Newsl..

[8] Paixão E.S., Teixeira M.G., Rodrigues L.C. (2018). Zika, chikungunya and dengue: The causes and threats of new and re-emerging arboviral diseases. BMJ Glob. Health.

[9] Gubler D.J. (2011). Dengue, Urbanization and Globalization: The Unholy Trinity of the 21st Century. Trop. Med. Health.

[10] Wilder-Smith A., Gubler D.J. (2008). Geographic Expansion of Dengue: The Impact of International Travel. Med. Clin. N. Am..

[11] Dick O.B., Martín J.L.S., Del Diego J., Montoya R.H., Dayan G.H., Zambrano B. (2012). The History of Dengue Outbreaks in the Americas. Am. J. Trop. Med. Hyg..

[12] Halstead S.B. (2006). Dengue in the Americas and Southeast Asia: Do they differ?. Rev. Panam. Salud Pública.

[13] Martín J.L.S., Brathwaite O., Zambrano B., Solórzano J.O., Bouckenooghe A., Dayan G.H., Guzmán M.G. (2010). The Epidemiology of Dengue in the Americas Over the Last Three Decades: A Worrisome Reality. Am. J. Trop. Med. Hyg..

[14] Adde A., Roucou P., Mangeas M., Ardillon V., Desenclos J.-C., Rousset D., Girod R., Briolant S., Quenel P., Flamand C. (2016). Predicting Dengue Fever Outbreaks in French Guiana Using Climate Indicators. PLoS Negl. Trop. Dis..

[15] Flamand C., Quenel P., Ardillon V., Carvalho L., Bringay S., Teisseire M. (2011). The Epidemiologic Surveillance of Dengue-Fever in French Guiana: When Achievements Trigger Higher Goals. Stud. Health Technol. Inform..

[16] Flamand C., Fritzell C., Prince C., Abboud P., Ardillon V., Carvalho L., Demar M., Boukhari R., Papaix-Puech M., Elenga N. (2017). Epidemiological assessment of the severity of dengue epidemics in French Guiana. PLoS ONE.

[17] Flamand C., Fritzell C., Matheus S., Dueymes M., Carles G., Favre A., Enfissi A., Adde A., Demar M., Kazanji M. (2017). The proportion of asymptomatic infections and spectrum of disease among pregnant women infected by Zika virus: Systematic monitoring in French Guiana, 2016. Eurosurveillance.

[18] Fouque F., Reynes J.-M., Moreau J.P. (1995). Dengue in French Guiana, 1965–1993. Bull. Pan Am. Health Organ..

[19] Fritzell C., Raude J., Adde A., Dusfour I., Quenel P., Flamand C. (2016). Knowledge, Attitude and Practices of Vector-Borne Disease Prevention during the Emergence of a New Arbovirus: Implications for the Control of Chikungunya Virus in French Guiana. PLoS Negl. Trop. Dis..

[20] Flamand C., Fabregue M., Bringay S., Ardillon V., Quénel P., Desenclos J.-C., Teisseire M. (2014). Mining local climate data to assess spatiotemporal dengue fever epidemic patterns in French Guiana. J. Am. Med. Inform. Assoc..

[21] L’Azou M., Taurel A.-F., Flamand C., Quénel P. (2014). Recent Epidemiological Trends of Dengue in the French Territories of the Americas (2000–2012): A Systematic Literature Review. PLoS Negl. Trop. Dis..

[22] Reynes J.-M., Laurent A., Deubel V., Telliam E., Moreau J.P. (1994). The First Epidemic of Dengue Hemorrhagic Fever in French Guiana. Am. J. Trop. Med. Hyg..

[23] Reynes J.-M. (1996). Dengue in French Guiana. History and present status. Bull. Soc. Pathol. Exot..

[24] Flamand C., Fritzell C., Obale P., Quenel P., Raude J. (2017). The Role of Risk Proximity in the Beliefs and Behaviors Related to Mosquito-Borne Diseases: The Case of Chikungunya in French Guiana. Am. J. Trop. Med. Hyg..

[25] Llagonne-Barets M., Icard V., Leparc-Goffart I., Prat C., Perpoint T., André P., Ramière C. (2016). A case of Mayaro virus infection imported from French Guiana. J. Clin. Virol..

[26] Talarmin A., Kazanji M., Bourreau E., Shope R.E., Lelarge J., LaBeau B., De Thoisy B., DeBon P., Vié J.C., Sarthou J.L. (1998). Mayaro virus fever in French Guiana: Isolation, identification, and seroprevalence. Am. J. Trop. Med. Hyg..

[27] De Alwis R., Williams K.L., Schmid M.A., Lai C.-Y., Patel B., Smith S.A., Crowe J., Wang W.-K., Harris E., De Silva A.M. (2014). Dengue Viruses Are Enhanced by Distinct Populations of Serotype Cross-Reactive Antibodies in Human Immune Sera. PLoS Pathog..

[28] Salje H., Paul K.K., Paul R., Rodriguez-Barraquer I., Rahman Z., Alam M.S., Rahman M., Al-Amin H.M., Heffelfinger J., Gurley E. (2019). Nationally-representative serostudy of dengue in Bangladesh allows generalizable disease burden estimates. eLife.

[29] Salje H., Cummings D.A.T., Rodríguez-Barraquer I., Katzelnick L.C., Lessler J., Klungthong C., Thaisomboonsuk B., Nisalak A., Weg A., Ellison D. (2018). Reconstruction of antibody dynamics and infection histories to evaluate dengue risk. Nat. Cell Biol..

[30] Fritzell C., Rousset D., Adde A., Kazanji M., Van Kerkhove M.D., Flamand C. (2018). Current challenges and implications for dengue, chikungunya and Zika seroprevalence studies worldwide: A scoping review. PLoS Negl. Trop. Dis..

[31] Flamand C., Bailly S., Fritzell C., Berthelot L., Vanhomwegen J., Salje H., Paireau J., Matheus S., Enfissi A., Fernandes-Pellerin S. (2019). Impact of Zika Virus Emergence in French Guiana: A Large General Population Seroprevalence Survey. J. Infect. Dis..

[32] Insee—National Institute of Statistics and Economic Studies. https://www.insee.fr/en/accueil.

[33] Beck C., Desprès P., Paulous S., Vanhomwegen J., Lowenski S., Nowotny N., Durand B., Garnier A., Blaise-Boisseau S., Guitton E. (2015). A High-Performance Multiplex Immunoassay for Serodiagnosis of Flavivirus-Associated Neurological Diseases in Horses. BioMed Res. Int..

[34] Cao-Lormeau V.-M., Blake A., Mons S., Lastère S., Roche C., Vanhomwegen J., Dub T., Baudouin L., Teissier A., Larre P. (2016). Guillain-Barré Syndrome outbreak associated with Zika virus infection in French Polynesia: A case-control study. Lancet.

[35] Hozé N., Salje H., Rousset D., Fritzell C., Vanhomwegen J., Bailly S., Najm M., Enfissi A., Manuguerra J.-C., Flamand C. (2020). Reconstructing Mayaro virus circulation in French Guiana shows frequent spillovers. Nat. Commun..

[36] Schiewe J. (2019). Empirical Studies on the Visual Perception of Spatial Patterns in Choropleth Maps. KN J. Cartogr. Geogr. Inf..

[37] StataCorp (2017). Stata Statistical Software: Release 15.

[38] QGIS Development Team QGIS 2 (2009). 18 Geographic Information System.

[39] Fouque F., Carinci R. (1996). Aedes aegypti in French Guiana. Some aspects of history, general ecology and vertical transmission of the dengue virus. Bull. Soc. Pathol. Exot..

[40] Epelboin Y., Chaney S.C., Guidez A., Habchi-Hanriot N., Talaga S., Wang L., Dusfour I. (2018). Successes and failures of sixty years of vector control in French Guiana: What is the next step?. Memórias Inst. Oswaldo Cruz.

[41] Hallet E., Flamand C., Rousset D., Bonifay T., Fritzell C., Matheus S., Dueymes M., Ntab B., Nacher M. (2020). ZIKA Virus infection in pregnant women in French Guiana: More precarious-more at risk. PLoS Negl. Trop. Dis..

[42] Dusfour I., Zorrilla P., Guidez A., Issaly J., Girod R., Guillaumot L., Robello C., Strode C. (2015). Deltamethrin Resistance Mechanisms in Aedes aegypti Populations from Three French Overseas Territories Worldwide. PLoS Negl. Trop. Dis..

